# Malarial Complication of Valvular Disease of the Heart

**Published:** 1874-07-01

**Authors:** Lester Curtis


THE
### MEDICAL EXAMINER.
A
Semi-Monthly Journal of Medical Sciences.

EDITED BY N. S. DAVIS, M.D., AND F. H. DAVIS, M.D.

No. XIII. CHICAGO, JULY 1, 1874. VOL. XV.

### Original Communications.

### MALARIAL COMPLICATION OF VALVULAR DISEASE OF THE HEART.

A CASE REPORTED BY LESTER CURTIS, M.D.

ABOUT two months since a robust Irishman, whom I had been fortunate in relieving of an asthmatic trouble, brought a young man to me for treatment, hoping that I might do something for him also, as the young man's trouble was, he stated, just like his own.

D—— H——, the new patient, was about sixteen years of age; he was pale and waxy looking, his lips only showing traces of color. He was not, however, emaciated, but had a puffy look about the face, and some œdema of the ankles.

He told me that he had been suffering from hæmorrhage of the bowels for two or three years: the attacks coming every two or three weeks or sometimes oftener. The hæmorrhages would come on without any premonition, he often not knowing of their occurrence until he saw the blood. The bleeding was often considerable in amount, and weakened him a good deal. With the exception of this trouble he claimed to have been well until about six months previous, when he began to experience difficulty of breathing after exertion. The shortness of breathing had come on gradually, and, at the time when I saw him, was so great that he could scarcely walk half a block without stopping to rest. I was unable, by the closest questioning, at his first or subsequent visits, to discover anything from which this dyspnœa could have arisen. He had never had any pain or swelling in the joints, or any symptoms corresponding to rheumatism. He had, when first examined, a dry, harsh cough, which dated like the dyspnœa to a period about six months previous, before which time he had never been troubled with a cough, nor had he ever been subject to any hard labor or strain of any kind.

Examination showed hæmorrhoidal tumors as the source of the bleeding from the rectum. Nothing abnormal could be detected about the lungs except a few rales.

The apex of the heart was in the sixth intercostal space, and somewhat to the left of the nipple. The lower border of the heart lay along the sixth intercostal space, and extended about an inch to the right of the sternum. The apex beat of the heart was not confined to one spot, but a wavy impulse extended over almost the whole of the space of dullness occupied by the heart.

A faint murmur, heard with difficulty and leaving room for considerable doubt as to its existence, could be heard at the apex. No other sound could be detected, but a pulsation in the jugulars also indicated tricuspid insufficiency. After a few days' treatment with digitalis and iron the extent of the dullness of the heart diminished perceptibly in size, then a systolic murmur at the apex came out loud and clear. From this I inferred that the left ventricle had been covered, and its sounds had been masked by a dilated right ventricle, and that the tonic action of the digitalis and iron, causing contraction of the right ventricle, had uncovered the apex of the left ventricle, and also that perhaps the sounds had become louder by the more vigorous contraction of the heart.

The patient continued steadily to improve, his hæmorrhoids ceased to bleed, the œdema disappeared, his color was better, and he became strong enough to walk a mile or more without uncomfortable fatigue or difficulty of breathing.

Wednesday morning, May 27, a message was sent to me to come to the patient immediately, as they considered him to be dying. On arriving at the house I was detained a few minutes by the priest, who had also been called to see him. When admitted I found him in a collapsed condition and laboring to breathe; his skin was pale and clammy, his face anxious, and dark circles surrounded his eyes; his pulse was feeble and fluttering, and his tongue covered with a yellow fur.

On inquiry, I learned that, the morning before, he had ridden down town without taking his breakfast, in company with an uncle and some boys; that he had staid away from home nearly the whole of the day, eating little and drinking several glasses of beer. He returned, however, without being intoxicated; but about three o'clock in the morning he awoke with a feeling of nausea, and soon began vomiting. He had vomited five or six times before I saw him, about half past seven o'clock; his bowels, however, had not moved, although he had once or twice seated himself upon the chamber with the expectation that they would move. His mother at first told me that he had been feeling well until the previous morning, but after careful inquiry I found that for a day or two his appetite had been poor and his bowels irregular: at one time relaxed, at another constipated, and that the passages had been of a greenish color and of a bad odor.

I prescribed some powders containing three grains of oxalate of cerium, to be given every two hours as long as the vomiting continued, and directed the following prescription to be given after an hour:

℞ Tr. Digitalis, f ʒ iij

Acidi Carbolici, gtt xij

Aquæ, f ℥ i

Glycerini, ad f ℥ ij

Mix. Sig. Teaspoonful every three hours.

I saw the patient again a little after noon. He had taken one of the powders at eight and one at ten, when the vomiting having ceased, they were stopped. He had also taken a teaspoonful of the digitalis mixture at nine o'clock and one at twelve. His bowels had moved soon after I left in the morning; the passage consisted of two or three hard greenish lumps. His skin was hot and dry; pulse, 120 per minute, and strong; the digitalis mixture was continued. I saw him again between seven and eight in the evening. He had taken the digitalis mixture at three and six. His pulse was softer and less frequent, and his skin was quite moist. I gave the mother ten sugar-coated pills of sulphate of quinine, directing her to give three pills at once, three at nine o'clock, and the remaining four at five o'clock the next morning.

Thursday—Saw him again at eight o'clock. He had rested well and was feeling well, except slight nausea. I prescribed eighteen grains of sulphate of quinine to be divided into six powders, one to be taken immediately, one before dinner, one before supper, and one at bed time.

Friday—Saw him again about eight in the morning. The mother had misunderstood the directions, and omitted the powder at bed time. He felt well, however. His bowels had not moved since Wednesday. I prescribed pil. hydrarg., gr. v, to be taken at once, and, if the bowels did not move, to be followed by a dose of epsom salts; quinine powders to be continued three times a day. The father came around to see me in the evening. He told me that the bowels had not moved, but the boy was feeling well. I directed small doses of quinine and iron to be taken for several days, and also ten drops of tincture of digitalis three times a day, to be continued indefinitely, and discontinued my visits.

